# Weight gain and metabolic disorders induced by psychotropic drugs: an appraisal of risk factors

**DOI:** 10.1192/j.eurpsy.2023.2086

**Published:** 2023-07-19

**Authors:** C. B. Eap

**Affiliations:** Département de Psychiatrie, Centre des Neurosciences Psychiatriques, Prilly, Switzerland

## Abstract

**Introduction:**

Weight gain and obesity are important health problems associated with psychiatric disorders and/or with psychotropic drug treatments. There is a high inter-individual variability in the susceptibility to drug induced weight gain and/or other cardiometabolic disorders.

**Objectives:**

To study the genetic, clinical and environmental risk factors for weight gain and onset of metabolic syndrome during psychotropic treatment.

**Methods:**

Analysis in PsyMetab, a large (n>3000) ongoing longitudinal prospective cohort study investigating cardiometabolic disorders in psychiatric patients.

**Results:**

Aside from well-known clinical risk factors for metabolic worsening (e.g. young age, first episode status, rapid weight gain during the first month of treatment and/or low initial BMI), we recently identified additional risk factors, such as the socio-economic status, a low status being associated with increased worsening of cardiometabolic parameters. Results from ongoing studies on the moderate dose dependencies of the metabolic effects of antipsychotics will be shown, as well as the clinical consequences. An epigenome-wide association study (EWAS) performed in 78 patients before and after one month of treatment (Dubath et al., submitted) and a genome-wide association study (GWAS) in 1924 patients (Sjaarda et al. submitted) will also be presented, as well as the use of polygenic risk scores to predict patients at risks for dyslipidemia (Delacretaz-Reymond et al., in preparation)

**Conclusions:**

Many factors contribute to the differences in weight gain and metabolic disorders induced by psychotropic drugs. The use of specific algorithms and/or polygenic risk scores can help to identify patients at risks. However, when starting a psychotropic drug at risk, a prospective monitoring of clinical (e.g. weight and blood pressure) and biochemical (fasting glucose, lipid levels) parameters is essential.

**Disclosure of Interest:**

None Declared

